# Microencapsulated *Ganoderma lucidum* extract improves antioxidant capacity, intestinal morphology, and nutrient digestibility in free-range laying hens under tropical conditions

**DOI:** 10.14202/vetworld.2025.3684-3697

**Published:** 2025-12-07

**Authors:** Tonglian Buwjoom, Piyaphat Petwattanapha, Buaream Maneewan, Pricha Rattanang, Wilmer Javier Pacheco, Sureerat Thuekeaw

**Affiliations:** 1 Faculty of Animal Science and Technology, Maejo University, Chiang Mai, 50290, Thailand; 2 Faculty of Agricultural Production, Maejo University, Chiang Mai, 50290, Thailand; 3 Department of Poultry Science, College of Agriculture, Auburn University, Auburn, AL 36849, USA

**Keywords:** antioxidant capacity, *Ganoderma lucidum*, intestinal morphology, laying hens, microencapsulation, nutrient digestibility, tropical heat stress

## Abstract

**Background and Aim:**

Free-range laying hens raised under tropical climates are susceptible to heat stress (HS), which compromises intestinal integrity, nutrient absorption, and egg quality. Ganoderma lucidum (Lingzhi) contains potent antioxidant compounds, but its efficacy in animal diets is limited by poor solubility and stability. This study evaluated the effects of dietary microencapsulated G. lucidum extract (MGE) on laying performance, antioxidant capacity, intestinal morphology, and nutrient digestibility in free-range hens under high-temperature conditions.

**Materials and Methods:**

A total of 256 Hy-Line Brown hens (25 weeks old) were randomly assigned to four dietary treatments with four replicates of 16 birds each: (i) basal diet (control [Con]), (ii) basal diet + free *G. lucidum* extract (1, 000 mg/kg), (iii) MGE at 1, 000 mg/kg (MGE0.1), and (iv) MGE at 500 mg/kg (MGE0.05). The trial lasted 12 weeks under natural tropical temperatures. Productive performance, egg quality, fatty acid profile, jejunal histomorphology, and antioxidant indices were analyzed. Statistical analysis was conducted using one-way analysis of variance with Tukey’s test (p ≤ 0.05).

**Results:**

MGE supplementation improved average egg weight and albumen height without affecting feed intake or feed conversion ratio. Both MGE0.1 and MGE0.05 increased jejunal villus height, width, surface area, and the villus height-to-crypt depth ratio compared with the Con. group. MGE diets significantly increased apparent ileal digestibility of dry matter, crude protein, gross energy, and ash. Enhanced antioxidant responses were observed, including higher total antioxidant capacity (15%–19%), superoxide dismutase (15%–22%), and catalase activity, with a concurrent 46%–47% reduction in malondialdehyde. Additionally, MGE diets reduced yolk cholesterol and the n-6/n-3 polyunsaturated fatty acid ratio.

**Conclusion:**

MGE effectively enhances intestinal morphology, antioxidant defense, and nutrient utilization in free-range hens exposed to HS, thereby improving egg quality and the yolk lipid profile. MGE represents a promising natural antioxidant and feed additive for sustainable poultry production under tropical conditions.

## INTRODUCTION

The global egg industry has undergone a substantial transformation as the adoption of organic, antibiotic-free, and free-range production systems has grown. This shift is largely driven by consumer awareness of human health, animal welfare, and environmental sustainability [1]. Surveys across 14 countries, including Australia, Bangladesh, Brazil, Chile, China, India, Malaysia, Nigeria, Pakistan, the Philippines, Sudan, Thailand, the United Kingdom (UK), and the United States (USA), indicate that most consumers prefer cage-free or organic eggs [2]. Despite this increasing demand, such production systems remain limited in practice, particularly in tropical regions, due to a lack of research aimed at improving production efficiency under challenging environmental conditions.

Free-range farming systems allow birds outdoor access and space to express natural behaviors [3]. However, these systems face multiple constraints, including exposure to pathogenic microorganisms, poor biosecurity, and heat stress (HS), all of which increase disease susceptibility, reduce productivity, and compromise egg and meat quality, thereby affecting overall farm profitability [4]. Because HS is known to impair intestinal integrity and nutrient absorption, strategies to preserve gut barrier function under stress are essential for maintaining animal health and performance [5].

Phytogenic feed additives (PFAs) have gained recognition as effective natural alternatives to antibiotic growth promoters (AGPs) [6]. In addition to enhancing intestinal health and performance, PFAs can replace synthetic antioxidants such as butylated hydroxyanisole and butylated hydroxytoluene, which raise safety concerns due to possible tissue accumulation and human health risks [7]. *Ganoderma lucidum* (Lingzhi), a medicinal mushroom belonging to the Ganodermataceae family, contains bioactive compounds, including polyphenols, terpenoids, sterols (e.g., ergosterol), and β-glucans, that exhibit potent antioxidant, anti-inflammatory, and antimicrobial properties [8]. The hydroalcoholic extract of *G. lucidum* demonstrates strong free radical scavenging activity, with inhibition rates up to 85% [9], and has been shown to reduce hepatic lipid accumulation in both mice [10] and laying hens [11].

However, the practical application of *G. lucidum* in poultry feed is constrained by the thermal and oxidative degradation of its bioactive constituents during processing and storage, as well as poor solubility and stability in the gastrointestinal (GI) tract. Enhancing its stability and targeted delivery through formulation technology is therefore crucial. Microencapsulation, a process that encloses active ingredients within a protective matrix, has emerged as a promising solution. This technology not only improves the physicochemical stability and controlled release of bioactive compounds but also enhances their bioavailability and antioxidant properties. For instance, microencapsulation has been reported to increase the antioxidant stability of *Myrtus communis* fruit extracts [12] and to improve the health and egg quality of poultry when applied to essential oils [13]. Similarly, liposomal encapsulation of oregano, cinnamon, and clove oils has demonstrated enhanced antioxidant, antibacterial, and gut-protective effects, as well as improved digestive enzyme activity in broiler chickens [14].

Despite extensive research on HS and the physiological responses of laying hens, relatively few studies have focused on developing effective, natural dietary strategies to sustain productivity in free-range systems within tropical climates. Free-range hens are particularly vulnerable to environmental stressors due to increased exposure to pathogens, fluctuating temperatures, and ultraviolet radiation, all of which impair gut integrity and oxidative balance. Although PFAs have been widely explored as alternatives to AGPs, most studies have utilized unencapsulated or thermolabile forms, which often show limited bioavailability, rapid degradation, and inconsistent physiological outcomes under field conditions.

*G. lucidum* (Lingzhi) is well-recognized for its strong antioxidant, immunomodulatory, and hepatoprotective effects in mammals and poultry. However, the thermosensitivity and poor solubility of its bioactive compounds, such as triterpenoids, ergosterols, and polyphenols, restrict its absorption and stability in feed matrices and the GI tract. Previous research has shown that *G. lucidum* extracts (GEs) can enhance antioxidant enzyme activity and reduce lipid accumulation in poultry, but their delivery efficiency and intestinal targeting remain suboptimal. Moreover, there is a scarcity of studies examining the use of microencapsulation technology to stabilize GEs and to evaluate its effects on gut histomorphology, nutrient digestibility, and egg lipid profile in hens maintained under tropical HS conditions.

This knowledge gap underscores the need for innovative feed technologies that can protect, stabilize, and deliver bioactive compounds effectively within the avian GI tract, thereby improving both bird welfare and production efficiency in environmentally challenging systems.

The present study was designed to investigate the potential of microencapsulated *G. lucidum* extract (MGE) as a natural antioxidant feed additive for free-range laying hens raised under tropical HS conditions. Specifically, this research aimed to:

1. Evaluate the effects of dietary supplementation with MGE at different inclusion levels on laying performance and egg quality.
2. Assess the influence of MGE on intestinal morphology and nutrient digestibility, with emphasis on jejunal villus architecture and apparent ileal digestibility (AID) of major nutrients.
3. Determine the impact of MGE supplementation on antioxidant enzyme activities, including total antioxidant capacity (T-AOC), superoxide dismutase (SOD), catalase (CAT), and malondialdehyde (MDA), in intestinal tissues.
4. Analyze changes in the fatty acid composition and cholesterol concentration in egg yolk to understand how MGE modulates yolk lipid metabolism and health-related egg traits.

By addressing these objectives, the study seeks to determine whether microencapsulation enhances the bioavailability and functional efficacy of *G. lucidum* compared with its free extract. The findings aim to provide scientific evidence supporting the use of MGE as a sustainable, natural alternative to improve antioxidant defense, gut health, and egg quality in free-range poultry production in tropical environments.

## MATERIALS AND METHODS

### Ethical approval

All animal procedures were conducted at the Faculty of Animal Science and Technology, Maejo University (Chiang Mai, Thailand). The experimental protocol was reviewed and approved by the Institutional Animal Care and Use Committee of Maejo University (Approval No. MACUC 016A/2565), following the ethical guidelines of the National Research Council (NRC) of Thailand. This study adhered to the Animal Research: Reporting of *In Vivo* Experiments guidelines and complied with the World Organization for Animal Health standards for the care and use of animals in research.

### Study period and location

The study was conducted from March to December 2023 at the Faculty of Animal Science and Technology, Maejo University, Chiang Mai, Thailand.

### Preparation of feed additives

Low-grade GE was procured from the Center of Lingzhi Mushroom Development and Organic Medicinal Mushroom, Faculty of Agricultural Production, Maejo University. The extract was microencapsulated using sodium alginate (SA) and chitosan (CS) as coating materials to form MGE. The resultant microcapsules had a mean particle diameter ranging from 800 to 1, 000 µm and an encapsulation efficiency exceeding 70%. Under simulated digestion conditions, MGE exhibited a controlled release of more than 80% with high thermal stability, as described by Petwattanapha *et al*. [15].

Free *G. lucidum* extract (FGE) was mixed with silicon dioxide in a 1: 2.5 ratio (extract: carrier) to ensure uniform dispersion in the feed. All reagents used were of analytical grade. The phytochemical composition and antioxidant potential of the *GE* are summarized in Supplementary Table S1.

### Experimental animals, design, and management

A total of 256 Hy-Line Brown laying hens, 18 weeks of age, were obtained from a commercial farm (Rungthongkhamfarm Co., Ltd., Chiang Mai, Thailand). Following an 8-week adaptation period on a basal diet (control [Con]), during which egg production stabilized at approximately 50%, birds aged 25 weeks were randomly allocated to four dietary treatments with four replicates per treatment (16 hens/replicate). The experimental treatments included:

- Con – basal diet without additives.
- FGE – basal diet + FGE (1, 000 mg/kg).
- MGE0.1 – basal diet + MGE (1, 000 mg/kg).
- MGE0.05 – basal diet + MGE (500 mg/kg).

The sample size was determined using G*Power software (version 3.1.9.4, Heinrich-Heine-Universität Düsseldorf, Düsseldorf, Germany), ensuring a minimum of 14 birds per group. To minimize bias, trained personnel who were blinded to the treatments conducted all experimental procedures. A schematic representation of the experimental design is shown in Supplementary Figure S1.

### Housing and environmental conditions

Each replicate was housed in a pen consisting of an indoor area (2 × 3 m) and an outdoor area (2 × 10 m), providing 1.625 m² per bird. The floor was covered with rice husk litter (3–5 cm thick). Feeders, drinkers, and two nest boxes were provided per pen. Clean tap water and feed were available *ad libitum*.

The basal diet was formulated to meet the nutrient requirements of laying hens according to the NRC [16] and the Hy-Line Brown Management Guide [17]. The feed ingredients and chemical compositions of the diets are shown in Table 1. The feed was prepared using a double-ribbon mixer for 3 min and stored at room temperature (~28°C) for up to 7 days. Birds were fed twice daily (07: 00 and 15: 00).

**Table 1:** Ingredient and nutrient composition of experimental diets fed to laying hens for 25–36 was of age (as-fed basis).

Ingredients (%) as-fed	Content
Corn	61.0
Soybean meal, 44% CP	24.1.
Fish meal, 55% CP	3.0
Limestone	7.0
Salt	0.3
Dicalcium phosphate, 18% P	3.0
DL-Methionine	0.3
L-Lysine	0.5
L-Threonine	0.2
Choline chloride 60%	0.5
Premix-layer hen^1)^	0.3
FGE or MGE^2)^	±

**Calculated nutrients, % as-fed**	

ME (kcal/kg)	2,975.6
CP	17.5
CF	4.0
EE	5.5
Calcium	3.7
Total phosphorus	0.9
Available phosphorus	0.2
Methionine	0.5
Lysine	1.2
Methionine + cystine	0.8
Tryptophan	0.1
Threonine	0.7
Sodium	0.2
Chloride	0.3

1) = Provided per kilogram of diet: vitamin A 9,900 IU, vitamin D3 4,000 IU, vitamin E 25 IU, vitamin K 32.5 IU, vitamin B1 2 mg, vitamin B2 6 mg, vitamin B6 4 mg, vitamin B12 0.024 mg, biotin 0.2 mg, pantothenic acid 10 mg, nicotinamide 35 mg, folacin 1 mg, choline 360 mg, Fe 80 mg, Cu 10 mg, Mn 100 mg, Zn 100 mg, 1.1 mg, Se 0.3 mg, and methionine 1.5 g. 2) FGE = Free Ganoderma *lucidum* extract, MGE = Microencapsulated Ganoderma *lucidum* extract, CP = Crude Protein, CF = Crude fiber, EE = Ether extract.

The study was conducted during the summer season (March–May), with morning and afternoon temperatures averaging 25°C and 35°C, respectively, and a relative humidity range of 80%-85%. Birds were exposed to 16 h of natural light and 8 h of incandescent illumination daily. Routine health monitoring and biosecurity protocols were implemented throughout the 12-week experimental period.

### Laying performance and egg quality evaluation

Feed intake, egg production, and mortality were recorded daily to calculate average daily feed intake (ADFI), feed conversion ratio (FCR), and hen-day production (HD%). These parameters were analyzed for three intervals: weeks 1–6, 7–12, and 1–12 of the experiment.

For egg quality assessment, 96 eggs (24 per treatment) were collected at 6-week intervals. Eggshell strength, thickness, and shape index were measured using a digital egg tester (DET6500, NABEL Co., Ltd., Kyoto, Japan). Albumen height and yolk diameter were used to calculate the Haugh unit (HU) and yolk index. Yolk color was determined using the Roche Yolk Color Fan (scale 1–15). The calculation procedure for HU is detailed in the supplementary materials.

### Analysis of fatty acid composition and cholesterol levels

For fatty acid profiling, 96 eggs (24/treatment) were collected over 3 consecutive days. Pooled yolk samples were homogenized, freeze-dried (CryoDry CD8, Australia), and analyzed through gas chromatography–mass spectrometry (GC–MS) (Model 4600, Unicom, UK) as per Peng *et al*. [18]. Lipid extraction was performed using a chloroform: methanol (2: 1, v/v) mixture, followed by phase separation with 0.88% NaCl and subsequent methylation.

The GC–MS system used a 5% diphenyl 95% dimethylpolysiloxane (DB-5) column (30 m × 0.25 mm), helium as the carrier gas (1 mL/min), and injector/detector temperatures of 250°C–300°C.Fatty acid methyl esters were identified using the Supelco 37 Component FAME Mix (TraceCERT®, Darmstadt, Germany), and results were expressed as mg/g of yolk.

The total fat and cholesterol contents were determined according to the method described by Folch *et al*. [19]. Dried yolk samples (2 g) were extracted with chloroform: methanol (60 mL) and purified through saponification with potassium hydroxide, followed by petroleum ether extraction. The supernatant was analyzed by high-performance liquid chromatography (Atlantis dC18 column, 4.6 × 250 mm, Waters, USA) using acetonitrile: methanol (60: 40, v/v) as the mobile phase (flow rate 2.0 mL/min; ultraviolet [UV] detection at 210 nm). Cholesterol quantification was based on an external calibration curve (R² = 0.9777) and expressed as mg/g of egg yolk.

### AID

During week 11, birds were fed diets containing titanium dioxide (TiO_2_, 5.0 g/kg) as an indigestible marker. At week 12, two hens per replicate were euthanized using CO_2_ inhalation, and ileal digesta were collected from Meckel’s diverticulum to the ileocecal junction. Samples were pooled per replicate, oven-dried, and analyzed for dry matter (DM), crude protein (CP), ether extract (EE), crude fiber (CF), gross energy (GE), and ash. GE was determined using a bomb calorimeter (Model AC-500, Leco, USA).

TiO_2_ concentration was analyzed as described by Short *et al*. [20]. Dried samples were ashed at 550°C for 4 h, dissolved in 7.4 M H_2_SO_4_ (10 mL), and oxidized with H_2_O_2_ (30%, 20 mL). Absorbance was measured at 410 nm using a UV/Vis spectrophotometer (Thermo Scientific, Evolution 201 series, USA). TiO_2_ recovery was 5.03 ± 0.1 g/kg. AID of nutrients was calculated using standard marker ratio equations.

### Jejunal histomorphology

At week 12, 2–3 cm segments of the mid-jejunum were collected, rinsed in phosphate-buffered saline, and fixed in 10% neutral-buffered formalin. Tissues were paraffin-embedded, sectioned, and stained with hematoxylin and eosin. Microscopic evaluation (40× magnification; Olympus BX5, Japan) was performed to measure villus height (VH), crypt depth (CD), and villus width (VW) from 10 points per sample. The villus height-to-crypt depth ratio (VH: CD) and villus surface area (VSA) were calculated. ImageJ software (National Institutes of Health, USA) and EPview software (EVIDENT Technology Center Europe GmbH, Münster, Germany) were used for morphometric analysis.

### Assessment of antioxidant capacity

Duodenal mucosa samples were collected, pooled per pen, and stored at −20°C. Homogenates were prepared using 0.05 M phosphate buffer (pH 7.4), centrifuged (1,500 × *g*, 15 min, 4°C), and the supernatant was analyzed for antioxidant indices: T-AOC, SOD, glutathione peroxidase (GSH-Px), CAT, and MDA.

T-AOC was determined by the 2, 2’-azino-bis (3-ethylbenzothiazoline-6-sulfonic acid (ABTS) assay [21] and expressed as µM Trolox equivalents. SOD activity was measured by inhibition of 5-amino-2, 3-dihydro-1, 4-phthalazinedione chemiluminescence, GSH-Px by nicotinamide adenine dinucleotide phosphate (NADPH) oxidation, and CAT by methanol-to-formaldehyde conversion in the presence of H2O2 [22]. MDA concentration was quantified using the thiobarbituric acid reactive substances method with 1,1,3,3-tetramethoxypropane as the calibration standard [23]. Intra-and inter-assay coefficients of variation were <5% and <10%, respectively.

### Statistical analysis

All experimental data were analyzed using a one-way analysis of variance (ANOVA) under the General Linear Model procedure in Statistical Analysis System (SAS) software version 9.4 (SAS Institute Inc., Cary, NC, USA). Each replicate pen was considered the experimental unit. Treatment means were compared using Tukey’s honestly significant difference test, and differences were regarded as statistically significant at *p* ≤ 0.05. Results are presented as mean values with their corresponding standard errors of the mean.

Before ANOVA, the Shapiro–Wilk test was employed to evaluate the normality of residuals, while Levene’s test was applied to confirm the homogeneity of variances. Effect sizes (η²) were reported for variables exhibiting significant treatment effects. A *post hoc* power analysis was conducted to ensure that the statistical power (1−β) exceeded 0.80 for the primary measured parameters.

The statistical model used for analysis was:

Yij = μ + Ti + eij

Where *Y*_ij_ represents the observation of the dependent variable, *μ* is the overall mean, *T*_i_ denotes the fixed effect of dietary treatment, and *e*_ij_ is the random error term.

## RESULTS

### Influence of dietary MGE on antioxidant capacity

**Figure 1:**
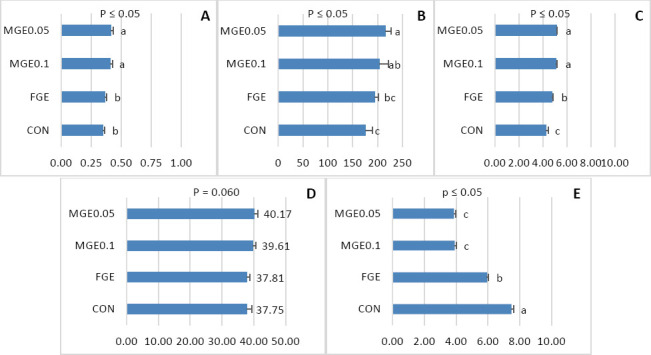
Antioxidant capacity of the duodenal mucosa in laying hens, including (A) Total antioxidant capacity in plasma. (B) Superoxide dismutase, (C) Catalase. (D) Glutathione peroxidase. (E) Malondialdehyde (n = 4 replicates). ^a–c^ Means with different superscripts are significantly different (p ≤ 0.05). Con, basal diet; FGE, basal diet with free *Ganoderma lucidum* extract at 1, 000 mg/kg; MGE0.1 and MGE0.05, basal diet with microencapsulated *Ganoderma lucidum* extract at 1, 000 and 500 mg/kg, respectively. Con = Control, FGE = Free *Ganoderma lucidum* extract, MGE = Microencapsulated Ganoderma lucidum extract.

Antioxidant parameters of the duodenal mucosa, including T-AOC, SOD, CAT, GSH-Px, and MDA, are presented in Figures 1a–e. Laying hens receiving MGE-supplemented diets (MGE0.1 and MGE0.05) showed significantly higher T-AOC levels (p < 0.05) compared with those fed the FGE and Con diets (Figure 1a). Dietary inclusion of MGE0.1 and MGE0.05 enhanced SOD activity by approximately 13% over the Con (p < 0.05), although no significant difference was observed between FGE and MGE0.1 treatments (Figure 1b).

The lowest CAT activity was detected in the Con group, whereas the highest value occurred in hens fed MGE0.05 (p < 0.05) (Figure 1c). GSH-Px activity exhibited an increasing trend at both MGE inclusion levels (p = 0.06) (Figure 1d). Additionally, MDA concentrations were markedly reduced by 46%–47% in hens receiving MGE diets compared with those fed the FGE and Con diets (p < 0.05) (Figure 1e). These results indicate that MGE supplementation effectively enhanced the antioxidant defense system and reduced oxidative damage in the intestinal mucosa.

### Effect of dietary MGE on jejunal histomorphology

**Figure 2:**
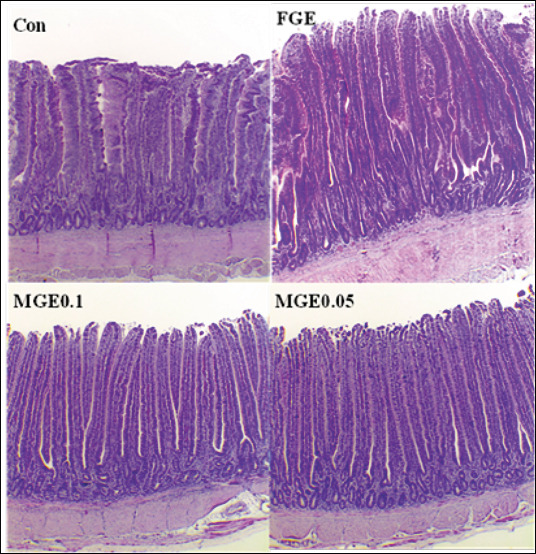
Representative morphology of the jejunum in laying hens fed the following diets: CON (basal diet), FGE (basal diet with free *Ganoderma lucidum* extract at 1,000 mg/kg), MGE0.1, and MGE0.05 (basal diet with microencapsulated *Ganoderma lucidum* extract at 1, 000 and 500 mg/kg, respectively). Two birds per replicate were analyzed, with 10 measurements per parameter on hematoxylin and eosin-stained sections at 40× magnification (XOS200). Con = Control, FGE = Free *G. lucidum* extract, MGE = Microencapsulated *Ganoderma lucidum* extract.

The histomorphological responses of the jejunum are summarized in Table 2 and illustrated in Figure 2. Dietary supplementation with either FGE or MGE significantly increased VH relative to the Con diet (p < 0.05). No significant differences in CD were observed among treatments (p > 0.05). Hens fed MGE diets exhibited greater VW and VSA than those fed Con (p < 0.05). Furthermore, both MGE0.1 and MGE0.05 diets significantly improved the VH: CD (p < 0.05), indicating enhanced intestinal absorptive capacity.

**Table 2:** Jejunum intestinal morphology of laying hens at 36 weeks of age^1)^.

Item	Experimental diets^2)^				SEM	p-value

	Con	FGE	MGE0.1	MGE0.5		
Villus height (µm)	979.00^c^	1,123.30^b^	1,258.02^a^	1,171.88^ab^	58.42	0.003
Crypt depth (µm)	177.06	173.97	155.42	143.78	7.88	0.299
Villus width (µm)	62.89^b^	76.60^a^	79.51^a^	80.99^a^	4.14	0.015
Villus height to the crypt depth ratio	4.94^c^	6.50^b^	8.30^a^	8.25^a^	0.80	<0001
Surface area of villus (mm^2^)	1.97^b^	2.71^a^	3.15^a^	2.98^a^	0.26	0.004

^a-c^Within a row, means with no common superscript differ significantly (p ≤ 0.05).

1) The means were calculated using 4 replicates per treatment.

2) Con, basal diet; FGE, basal diet with free *Ganoderma lucidum* extract at 1,000 mg/kg; MGE0.1 and MGE0.05, basal diet with microencapsulated *Ganoderma lucidum* extract at 1,000 and 500 mg/kg, respectively. Con = Control, FGE = Free *Ganoderma lucidum* extract, MGE = Microencapsulated *Ganoderma lucidum* extract, SEM = Standard errors of the mean.

### Influence of dietary MGE on AID

The effects of MGE supplementation on the AID of nutrients in laying hens at 36 weeks of age are presented in Table 3. The AID of DM was significantly improved by all additive treatments, FGE (10.39%), MGE0.1 (13.69%), and MGE0.05 (10.25%), compared with the Con group (p < 0.01). CP digestibility was also higher in the MGE0.1 group relative to the Con (p = 0.013). GE and ash digestibility were enhanced by all feed additives (p < 0.01), whereas no significant effects were observed for EE and CF (p > 0.05). These findings suggest that MGE supplementation improved nutrient utilization efficiency in laying hens.

**Table 3:** Apparent ileal digestibility of laying hens at 36 weeks of age^1)^.

Item	Experimental diets^2)^				SEM	p-value

	Con	FGE	MGE0.1	MGE0.05		
Dry matter (%)	58.96^b^	65.79^a^	68.31^a^	65.69^a^	2.00	0.001
Crude protein (%)	63.83^b^	74.35^a^	77.08^a^	73.12^ab^	2.88	0.013
Ether extract (%)	50.11	58.72	65.91	56.79	3.25	0.062
Crude fiber (%)	41.99	41.91	45.64	41.43	0.97	0.895
Gross energy (%)	60.30^b^	70.19^a^	71.27^a^	71.19^a^	2.66	0.001
Ash (%)	32.67^b^	38.83^a^	38.86^a^	37.78^a^	1.48	0.001

^a–c^Within a row, means with no common superscript differ significantly (p *≤* 0.05).

1) The means were calculated using 4 replicates per treatment.

2) Con, basal diet; FGE, basal diet with free *Ganoderma lucidum* extract at 1,000 mg/kg; MGE0.1 and MGE0.05, basal diet with microencapsulated *Ganoderma lucidum* extract at 1,000 and 500 mg/kg, respectively. Con = Control, FGE = Free Ganoderma *lucidum* extract, MGE = Microencapsulated *Ganoderma lucidum* extract, SEM = Standard errors of the mean.

### Effect of dietary MGE on laying performance

Performance parameters of laying hens are summarized in Table 4. During weeks 1–6, dietary antioxidants had no significant influence on ADFI, HD%, or FCR (p > 0.05). However, hens fed FGE, MGE0.1, or MGE0.05 diets exhibited significantly higher average egg weight (AEW) than those in the Con group (p = 0.013). Across the 7–12 and 1–12 week periods, ADFI showed an increasing trend in antioxidant-fed hens (p = 0.087 and 0.089, respectively), although HD% and FCR remained unaffected (p > 0.05). Over the entire 12-week trial, AEW remained significantly higher in all supplemented groups (p = 0.049), while mortality rates did not differ among treatments (p = 0.495).

**Table 4:** Effect of supplementation with microencapsulated *Ganoderma lucidum* extract on the performance of laying hens^1^ (25–36 weeks of age)^1)^.

Item	Experimental diet ^2)^				SEM	p-value

	Con	FGE	MGE0.1	MGE0.05		
Initial weight (kg)	1.72	1.75	1.80	1.77	0.017	0.426
1–6 weeks						
ADFI (g/d/hen)	102.54	104.31	105.70	102.78	0.737	0.391
%HD	82.33	88.99	86.68	81.66	1.756	0.331
AEW (g)	52.38^b^	54.81^a^	54.69^a^	54.95^a^	0.428	0.013
FCR (feed-to-eggs)	2.31	2.14	2.23	2.39	0.053	0.171
7–12 weeks						
ADFI (g/d/hen)	101.64	104.61	105.85	104.74	0.941	0.087
%HD	78.79	83.56	82.92	78.35	1.357	0.446
AEW (g)	54.11	54.56	53.73	52.10	0.536	0.198
FCR (feed-to-eggs)	2.39	2.31	2.38	2.53	0.046	0.403
Overall, 1-12 weeks						
ADFI (g/d/hen)	102.09	104.46	105.78	104.76	0.835	0.089
%HD	80.56	86.27	84.80	80.00	1.551	0.310
AEW (g)	52.26^b^	54.69^a^	54.23^a^	54.53^a^	0.476	0.049
FCR (feed-to-eggs)	2.35	2.22	2.30	2.45	0.049	0.257
Mortality rate (%)	4.69	3.13	1.56	1.56	0.748	0.495

^a,b^ Within a row, means with no common superscript differ significantly (p *<* 0.05).

1) Means were calculated using 4 replicates (16 hens/replicate) per treatment.

2) Con, basal diet; FGE, basal diet with free Ganoderma *lucidum* extract at 1,000 mg/kg; MGE0.1 and MGE0.05, basal diet with microencapsulated *Ganoderma lucidum* extract at 1,000 and 500 mg/kg, respectively. ADFI = Average daily feed intake, HD% = Hen day production, AEW = Average egg weight, FCR = Feed conversion ratio, Con = Control, FGE = Free *Ganoderma lucidum* extract, MGE = Microencapsulated *Ganoderma lucidum* extract, SEM = Standard errors of the mean.

### Effect of dietary MGE on egg quality

Egg quality parameters are presented in Table 5. At week 6, no significant effects of dietary treatments were observed on shell strength, shell thickness, shape index, yolk color, or yolk index (p > 0.05). However, albumen height was significantly higher in hens fed MGE0.1 and MGE0.05 than in those fed the Con (p = 0.028). The highest HU values were recorded in MGE0.05 (82.89) and MGE0.1 (82.61) diets, both of which were superior to FGE and Con groups (p = 0.001).

At week 12, MGE0.05 again yielded the highest albumen height (p = 0.026), while all additive treatments improved yolk index (p = 0.040). HU values ranged between 80 and 82 across treatments, with no significant differences among groups (p > 0.05). These results demonstrate that MGE supplementation enhanced internal egg quality, particularly albumen height and HU values.

### Influence of dietary MGE on fatty acid profile and yolk cholesterol

The fatty acid composition of egg yolk, including saturated fatty acids (SFAs), monounsaturated fatty acids (MUFAs), polyunsaturated fatty acids (PUFAs), and cholesterol levels, is shown in Table 6. Hens fed the MGE0.05 diet had significantly higher total SFAs, MUFAs, and PUFAs compared with those fed FGE (p < 0.001). The MGE0.1 group exhibited the highest eicosapentaenoic acid (C20: 5n-3) content, whereas MGE0.05 resulted in the highest docosahexaenoic acid (C22: 6n-3) concentration. Both MGE treatments increased total omega-3 (n-3) and omega-6 (n-6) PUFAs in the yolk, while simultaneously reducing the n-6/n-3 ratio compared with Con and FGE groups (p < 0.001).

**Table 5:** Effect of microencapsulated *G. lucidum* extract on egg quality^1)^.

Item	Experimental diets^2)^				SEM	p-value

	Con	FGE	MGE0.1	MGE0.05		
6 weeks						
Shell strength (N)	27.79	28.29	29.35	28.77	0.334	0.637
Shell thickness (mm)	0.41	0.42	0.40	0.43	0.008	0.415
Shape index	76.89	75.95	76.51	76.27	0.198	0.618
Albumen height (mm)	6.35^b^	6.72^ab^	7.19^a^	6.99^a^	0.181	0.028
Yolk color	7.21	7.33	7.21	6.88	0.098	0.088
Yolk index	0.27	0.26	0.29	0.39	0.030	0.309
Haugh unit	80.98^b^	79.94^b^	82.61^a^	82.89^a^	0.696	0.001
12 weeks						
Shell strength (N)	25.03	23.82	25.52	25.32	0.380	0.325
Shell thickness (mm)	0.34^ab^	0.36^a^	0.33^ab^	0.32^b^	0.008	0.050
Shape index	76.24	77.51	77.61	75.75	0.464	0.271
Albumen height (mm)	6.71^bc^	6.60^c^	6.95a^b^	7.04^a^	0.102	0.026
Yolk color	8.00	7.54	7.58	7.79	0.106	0.163
Yolk index	0.30^b^	0.33^a^	0.33^a^	0.33^a^	0.006	0.040
Haugh unit	80.93	80.42	81.81	82.12	0.390	0.247

^a–d^Within a row, means with no common superscript differ significantly (p ≤ 0.05).

1) The means were calculated using 4 replicates per treatment.

2) Con, basal diet; FGE, basal diet with free *Ganoderma lucidum* extract at 1,000 mg/kg; MGE0.1 and MGE0.05, basal diet with microencapsulated Ganoderma *lucidum* extract at 1,000 and 500 mg/kg, respectively. Con = Control, FGE = Free *Ganoderma lucidum* extract, MGE = Microencapsulated *Ganoderma lucidum* extract, SEM = Standard errors of the mean.

**Table 6:** Fatty acids in the yolk (mg/g of egg yolk)^1^.

Fatty acids	Experimental diet (mg/g)^2^				SEM	p-value

	Con	FGE	MGE0.1	MGE0.05		
Pentadecanoic (C15:0)	0.30^b^	0.40^a^	0.30^b^	0.40^a^	0.029	<0.001
Palmitic (C16:0)	140.95^a^	136.90^b^	126.55^c^	141.60^a^	3.476	<0.001
Heptadecanoic (C17:0)	0.80^b^	0.80^b^	0.70^c^	0.90^a^	0.041	<0.001
Stearic (C18:0)	41.35^b^	41.20^b^	36.70^c^	42.20^a^	1.241	<0.001
Total SFAs	185.30^a^	181.20^b^	166.05^c^	187.00^a^	4.770	<0.001
Palmitoleic (C16:1)	23.05^a^	22.60^b^	21.10^c^	23.00^a^	0.457	<0.001
Cis-9-Oleic (C18:1n9c)	223.35^b^	222.05^b^	199.65^c^	228.55^a^	6.406	<0.001
Cis-11-Eicosenoic (C20:1)	1.55^c^	1.60^b^	1.40^d^	1.70^a^	0.063	<0.001
Total MUFAs	248.94^b^	247.25^b^	223.15^c^	254.25^a^	6.912	<0.001
Linoleic acid (C18:2n-6)	58.20^b^	56.30^c^	55.05^c^	62.55^a^	1.641	<0.001
Gamma-linolenic acid (C18:3n-6)	0.60^a^	0.60^a^	0.50^b^	0.60^a^	0.025	<0.001
Alpha-linolenic (C18:3n-3)	1.70^b^	1.60^c^	1.50^d^	2.00^a^	0.108	<0.001
Cis-11,14-Eicosadienoic acid (C20:2n-6)	1.00^a^	0.80^b^	0.70^c^	0.80^a^	0.063	<0.001
Cis-8,11,14-Eicosadienoic acid (C20:3n-6)	1.20^a^	1.10^b^	1.00^c^	1.20^a^	0.048	<0.001
Arachidic acid (C20:4n-6)	9.10^b^	8.60^c^	8.10^d^	9.90^a^	0.384	<0.001
Eicosapentaenoic acid (C20:5n-3)	1.10^d^	1.15^c^	1.40^a^	1.30^b^	0.069	<0.001
Docosahexaenoic acid (C22:6n-3)	3.50^b^	3.30^d^	3.40^c^	4.30^a^	0.229	<0.001
Total PUFAs	76.40^b^	73.45^c^	70.00^d^	82.65^a^	2.682	<0.001
Total number of n-6 fatty acids	70.10^b^	67.40^c^	63.70^d^	75.05^a^	2.388	<0.001
Total n-3 fatty acid content	6.30^b^	6.05^c^	6.30^b^	7.60^a^	0.351	<0.001
Total n-6/n-3	111.27^a^	111.41^a^	101.13^b^	98.75^c^	3.326	<0.001
Total fat content (%)	52.98^b^	52.40^b^	48.15^c^	55.23^a^	14.799	<0.001
Total cholesterol (mg/g yolk)	22.24^b^	24.68^a^	21.42^c^	22.26^b^	0.705	<0.001

^a–d^Within a row, means with no common superscript differ significantly (p ≤ 0.05).

1 Means represent 24 eggs per treatment (4 replicates per treatment).

2 Con, basal diet; FGE, basal diet with free *Ganoderma lucidum* extract at 1,000 mg/kg; MGE0.1 and MGE0.05, basal diet with microencapsulated *Ganoderma lucidum* extract at 1,000 and 500 mg/kg, respectively. SFAs = Saturated fatty acids, MUFAs = Monounsaturated fatty acids, PUFAs = Polyunsaturated fatty acids, Con = Control, FGE = Free *Ganoderma lucidum* extract, MGE = Microencapsulated *Ganoderma lucidum* extract, SEM = Standard errors of the mean, ALA = Alpha-linolenic (C18:3n-3).

The total fat content in egg yolk ranged from 52% to 55%, whereas cholesterol concentrations varied between 21 mg/g and 24 mg/g. Notably, MGE0.1 supplementation reduced yolk cholesterol to 21.42 mg/g, the lowest among treatments (p < 0.001). Complete data are provided in Supplementary Table S2.

## DISCUSSION

### HS challenge and the role of microencapsulated antioxidants

Laying hens reared under tropical and subtropical conditions are frequently exposed to high ambient temperatures that induce chronic HS. The present experiment was conducted during the summer season, when environmental temperatures exceeded the thermal comfort range of laying hens [24]. Prolonged exposure to high temperature disrupts normal physiological homeostasis, impairs feed efficiency, and decreases productivity [25].

To counter these effects, this study evaluated the potential of dietary antioxidants formulated through microencapsulation to improve physiological resilience and productivity under HS. Microencapsulation technology protects bioactive compounds as they transit through the GI tract, allowing for controlled release at the target site. It also enhances thermostability, oxidative stability, and shelf life, while masking unpleasant flavors [26]. Thus, this approach offers a novel strategy for delivering heat-sensitive phytogenic compounds such as GEs in poultry feed.

### Antioxidant response and redox balance

Chronic HS elevates the generation of reactive oxygen and nitrogen species, including superoxide (O2•¯), hydroxyl (OH•), nitric oxide (NO•), and nitrogen dioxide (NO2•), which disrupts the cellular redox balance in poultry [27]. The antioxidant defense system comprises enzymatic components such as SOD, CAT, and GSH-Px, which neutralize radicals, and non-enzymatic antioxidants such as vitamins, phenolics, and plant phytochemicals that scavenge free radicals. T-AOC reflects the overall balance between oxidative stress and antioxidant defense.

In this study, supplementation with MGE significantly increased T-AOC, SOD, and CAT activities in the duodenal mucosa, demonstrating enhanced antioxidative status. This observation supports earlier findings that GEs upregulate antioxidant-related genes in the small intestine [28]. Simultaneously, MDA concentrations, a marker of lipid peroxidation, were markedly reduced in MGE-supplemented groups, indicating lower oxidative damage. The improvement can be attributed to the synergistic activity of ergosterol, triterpenoids, flavonoids, and phenolic compounds in *G. lucidum*, which act as electron donors to neutralize free radicals [29]. Similar reductions in MDA were reported by Wang *et al*. [30], who observed decreased plasma MDA in broilers fed *G. lucidum* at 300 mg/kg.

### Intestinal morphology and functional improvement

Oxidative stress can damage macromolecules, proteins, lipids, and nucleic acids, particularly in the intestinal epithelium, which plays a key role in nutrient absorption [31]. In the present study, hens fed the Con diet had the shortest VH and the lowest VW, VH: CD ratio, and VSA. In contrast, MGE supplementation markedly improved these parameters, indicating enhanced intestinal structure and absorptive capacity. Chen and Yu [28] similarly reported improved intestinal morphology in broilers receiving GE.

The enhanced jejunal histomorphology observed here likely results from the antioxidant constituents of *G. lucidum*, which protect epithelial cells from oxidative injury, stabilize tight junctions, and promote mucosal renewal. Improved intestinal integrity increases the surface area for nutrient uptake and facilitates greater digestive enzyme secretion, thereby improving feed utilization efficiency.

### Nutrient digestibility and metabolic utilization

The AID results revealed that supplementation with either FGE or MGE enhanced the digestibility of DM, CP, GE, and ash. This may be explained by improvements in villus morphology, which increase the nutrient-absorption surface area and enzymatic activity. Similar outcomes have been reported in laying hens supplemented with Agaricus bisporus stem residues, which enhanced total energy digestibility [32]. Moreover, *G. lucidum* compounds have been shown to upregulate digestion-related genes in Nile tilapia, promoting better nutrient utilization [33]. Therefore, microencapsulation appears to amplify the functional benefits of *G. lucidum* by ensuring more efficient delivery of its bioactive components within the GI tract.

### Laying performance and productive efficiency

In the early laying phase, supplementation with FGE or MGE did not significantly influence feed intake, egg production rate, or FCR, although egg weight improved significantly. These findings align with previous observations that *GE* did not alter broiler performance indices [28]. The slightly lower ADFI recorded in free-range hens compared with conventional systems is typical, as greater physical activity under outdoor conditions diverts energy from production [34]. Despite environmental fluctuations, egg production remained within a normal range, confirming that the supplemented diets met nutritional requirements. The observed increase in egg weight may be linked to improved nutrient digestibility (DM and GE) and enhanced metabolic efficiency resulting from MGE supplementation.

### Egg quality parameters

Egg quality directly affects marketability and consumer preference. In this study, MGE supplementation improved internal egg quality, reflected by higher albumen height, HU, and yolk index values, without altering shell strength or thickness. These improvements may be attributed to the antioxidant protection provided by MGE, which mitigates oxidative damage in reproductive tissues, particularly the magnum, which is responsible for albumen formation [5]. Additionally, phytogenic antioxidants slow down lipid peroxidation in yolk and albumen, maintaining freshness and texture [35]. Overall, these findings highlight that microencapsulated antioxidants support egg quality stability in hens exposed to HS.

### Egg yolk lipid profile and cholesterol modulation

MGE supplementation favorably altered yolk fatty acid composition and cholesterol concentration. The MGE0.05 diet enhanced total SFA, MUFA, and PUFA contents, while both MGE diets reduced the n-6/n-3 PUFA ratio, an indicator of improved lipid health and cardiovascular benefits [36]. Notably, the MGE0.1 diet lowered yolk cholesterol to 21.42 mg/g, suggesting hypocholesterolemic activity. This effect likely arises from *G. lucidum*’s triterpenoids, which inhibit hepatic 3-hydroxy-3-methylglutaryl-CoA reductase and enhance cholesterol catabolism and fecal excretion [37]. Similar cholesterol-lowering effects were reported in mice fed GEs [38].

While fatty acid variations in eggs may also depend on breed, age, and diet composition, the consistent experimental Con in the current study suggests that MGE supplementation was the major contributing factor. The soybean meal and corn-based basal diet provided adequate linoleic and oleic acids, which were likely modified by the bioactive compounds in MGE to enhance yolk lipid quality [39].

### Controlled release mechanism and functional implications

The superior performance of MGE compared with FGE may be explained by the controlled release behavior of its encapsulating materials, SA and CS. The CS matrix swells and gradually dissolves in the acidic stomach environment (pH 2–3) due to protonation of amine groups (–NH2), enabling slow diffusion of encapsulated actives [40]. SA resists acid hydrolysis and enzymatic degradation, protecting bioactives during upper GI transit. As the pH increases beyond 5 in the duodenum, the carboxyl groups (–COOH) of alginate ionize, leading to gradual dissolution and release of active compounds [41]. This mechanism ensures targeted delivery and improved bioavailability of *G. lucidum* constituents along the intestinal tract, contributing to the observed physiological benefits.

### Future perspectives

The present findings demonstrate that MGE effectively enhances intestinal health, antioxidant capacity, and yolk lipid quality in laying hens exposed to tropical HS. However, responses may vary depending on plant species, dosage, bird age, production system, and encapsulation characteristics. Future studies should evaluate the long-term effects of MGE across the full laying cycle, focusing on mechanisms of absorption, modulation of the gut microbiota, and reproductive performance. Such insights would advance the development of sustainable feed additives that improve productivity, welfare, and egg quality in free-range poultry systems.

## CONCLUSION

Supplementation of MGE significantly improved the physiological and production responses of free-range laying hens under tropical HS. Both MGE0.05 and MGE0.1 diets enhanced T-AOC, SOD, and CAT activities while reducing MDA levels by approximately 47%, indicating reduced oxidative damage. Improved VH, VSA, and VH: CD ratios were associated with higher AID for DM, CP, and GE. MGE supplementation also increased albumen height and HU scores, while decreasing yolk cholesterol and the n-6/n-3 PUFA ratio, reflecting improved internal egg quality and nutritional value.

GE can be applied as a natural, heat-stable antioxidant feed additive to enhance gut health, antioxidant defense, and egg quality in free-range hens raised under high-temperature environments. The improved yolk lipid profile and reduced cholesterol content increase the functional and consumer appeal of eggs, contributing to sustainable, antibiotic-free poultry production.

This study is among the first to evaluate MGE in free-range laying hens, combining physiological, biochemical, histological, and production-level analyses. The integration of microencapsulation technology ensured controlled bioactive release, enhanced stability, and greater bioavailability, providing a strong foundation for its commercial application.

The experiment was restricted to the early laying phase and short-term evaluation under specific tropical summer conditions. Long-term trials across different breeds, housing systems, and climatic seasons are necessary to confirm consistency, optimize dosage, and assess cost-effectiveness.

Microencapsulation of GE offers a promising and practical nutritional strategy to mitigate HS, enhance intestinal integrity, and improve egg quality in free-range poultry systems. These findings provide scientific support for the broader application of phytogenic encapsulated additives as sustainable alternatives to synthetic antioxidants and growth promoters in the poultry industry.

## DATA AVAILABILITY

All the generated data are included in the manuscript.

## AUTHORS’ CONTRIBUTIONS

ST: Conceptualization, methodology, validation, formal analysis, investigation, manuscript review and editing, and supervision. TB: Conceptualization, methodology, validation, data collection, and analysis. PP: Data collection and analysis. BM: Conceptualization and analysis. PR: Conceptualization and analysis. WJP: Conceptualization investigation; reviewed and edited the manuscript. All authors have read and approved the final version of the manuscript.
